# Protocol for the development, assembly, and testing of a synthetic skin microbial community

**DOI:** 10.1016/j.xpro.2025.103714

**Published:** 2025-03-25

**Authors:** Deepan Thiruppathy, Asama Lekbua, Joanna Coker, Yuhan Weng, Fatemeh Askarian, Armin Kousha, Clarisse Marotz, Amber Hauw, Megan Tjuanta, Victor Nizet, Karsten Zengler

**Affiliations:** 1 Division of Host-Microbe Systems & Therapeutics, Department of Pediatrics, University of California, San Diego, 9500 Gilman Drive, La Jolla, CA 92093-0760, USA; 2 Department of Bioengineering, University of California, San Diego, La Jolla, CA 92093-0412, USA; 3 School of Biological Sciences, University of California, San Diego, La Jolla, CA 92093-0376, USA; 4 Bioinformatics and Systems Biology Program, University of California, San Diego, La Jolla, CA 92093-0419, USA; 5 Glycobiology Research and Training Center, University of California, San Diego, La Jolla, CA, USA; 6 Skaggs School of Pharmacy and Pharmaceutical Sciences, University of California, San Diego, La Jolla, CA, USA; 7 Center for Microbiome Innovation, University of California, San Diego, La Jolla, CA 92093-0403, USA; 8 Program in Materials Science and Engineering, University of California, San Diego, 9500 Gilman Drive, La Jolla, CA 92093-0418, USA

**Keywords:** Bioinformatics, Microbiology, Model Organisms

## Abstract

A reproducible study system is essential for understanding the role of microbes in human skin health and disease. We present a protocol for constructing a synthetic microbial community (SkinCom) of nine strains dominant to native human skin microbiome. We describe steps for computing growth metrics, constructing communities, and extracting DNA and library preparation for shotgun sequencing. We detail steps for data preprocessing and analysis of community samples. We illustrate SkinCom’s application with an epicutaneous murine model and downstream multiomic analysis.

For complete details on the use and execution of this protocol, please refer to Lekbua et al.^1^

## Highlights

• Automated construction of a reproducible synthetic microbial community for human skin
• Steps for computing microbial growth metrics for nine dominant human-skin bacteria
• Extracting DNA for metagenomics and metatranscriptomics
• Computational pipeline steps for preprocessing and analyzing FASTQ sequencing files

## Abstract (editor highlights)

Publisher’s note: Undertaking any experimental protocol requires adherence to local institutional guidelines for laboratory safety and ethics.

## Graphical abstract

**Figure:**
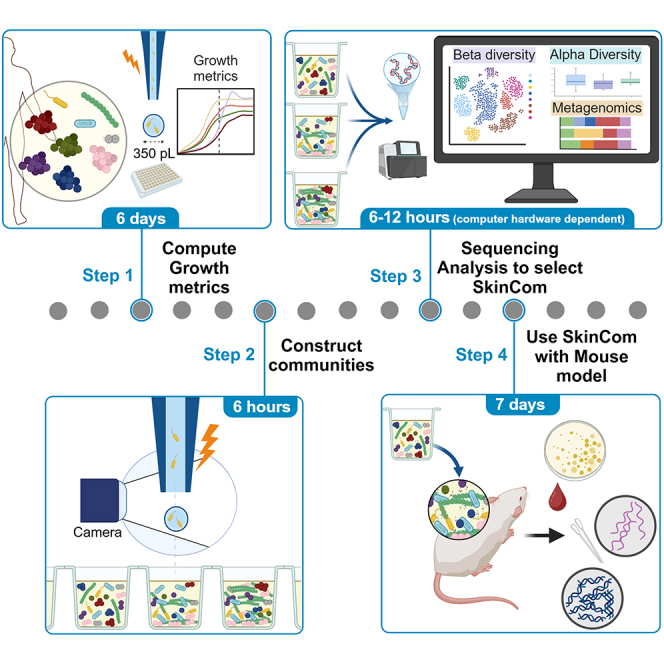

## Before you begin

1. Clearly label and date all consumables containing microbes.
2. Choose candidate microbes for the synthetic microbial community. The selected candidates should be abundant in the human skin microbiome characterized across multiple studies and represent the biome’s metabolic diversity (aerobic, microaerophilic, and anaerobic organisms; gram-positive and gram-negative). They must also grow on the same basal media mix.
3. Ensure all mice are housed in the same experimental room with the appropriate light/dark cycles and controlled for temperature and humidity.
4. Ensure you have access to the appropriate versions of R and corresponding packages (see key resources table for full list of packages and the required versions).

### Obtaining SkinCom strains and NCBI nucleotide reference sequences

**Timing: variable; depends on location and delivery method**

Strains of the human skin microbiome chosen for this study are listed below. The strains were selected for the representativeness in the skin microbiome based on prior literature reports,^2^^,^^3^^,^^4^ along with ease of procurement and propagation of all bacteria in the same media. For further details, see Lekbua et al.^1^

Corynebacterium afermentans (ATCC 51403, strain CIP 103499 [LCDC 88199]), Cutibacterium acnes (ATCC KPA171202), Micrococcus luteus (ATCC 4698), Staphylococcus capitis (ATCC 27840, strain LK 499), Staphylococcus epidermidis (ATCC 12228), Staphylococcus hominis (ATCC 27844, strain DM 122), Staphylococcus warneri (ATCC 27836, strain AW 25), Streptococcus mitis (ATCC 49456, strain NCTC 12261) and Staphylococcus aureus (ATCC 35556, strain SA113).

5. Purchase strains from American Type Culture Collection (ATCC, https://www.atcc.org/), or Leibniz Institute DSMZ-German Collection of Microorganisms and Cell Cultures GmbH (DSMZ, https://www.dsmz.de/).

***Note:*** Some strains are only available from ATCC.

6. Download the corresponding nucleotides of the complete genomes for the strains from repositories like NCBI, Ensembl, UCSC Genome Browser, etc.

### Institutional permissions

Animal experiments should be conducted in accordance with the rules and regulations of the Institutional Animal Care and Use Committee, which has been approved by the affiliated university. The steps in this protocol were approved by the UC. San Diego IRB protocol S00227M.

### Preparing anti-condensation lid for plate reading assays

**Timing: ∼40 min (10 min preparation, 30 min waiting)**

To mitigate evaporative losses that can cause inaccurate spectrophotometric readouts of microbial growth over time, replace regular 96-well plate lids with anti-condensate-coated plate lids.

7. Prepare 5 mL of anti-condensate mix: 0.05% Triton X-100 in 20% ethanol, filter sterilized.
8. Pour 3 mL of this mix into a fresh 96-well plate lid.

***Note:*** Ensure that the coating covers each of the depressions in the lid for all 96 wells in the plate. Tilt the lid down towards the bottom left and use a 1 mL pipette to collect the residue from the bottom and re-inject it back from the top to ensure an even coating across the entire lid.

**CRITICAL:** This step must be performed in a sterilized biosafety cabinet.

9. After ∼30 s, pour off the remaining treatment solution and shake off any leftover drops.

***Note:*** About 1.5–1.8 mL of the treatment solution should be recovered if sufficient coating was applied.

10. Lean the lid against a vertical surface to air dry.

**CRITICAL:** This step must be performed in a sterilized biosafety cabinet. Can leave the UV on in the safety cabinet during this step, following necessary precautions and PPE.

**Pause Point:** The lid needs at least 30 min to completely dry before it is ready to use.

### Preparing system fluid, wash bottle, and waste bottle for CellenONE X1 picoliter droplet dispenser

**Timing: ∼2 h**

The CellenONE X1 uses piezo-acoustic technology to produce pico-liter volumes of droplets at micrometer precision. This lends the robot for multiple uses, including for the construction of defined communities of microorganisms.^5^ The machine uses silicone capillary tubing with 1.5 mm diameter. The machine also uses peristaltic pumps and syringes to aspirate and dispense droplets through the Piezoelectric Dispensing Capillaries nozzles (PDC). It is essential that the liquid that runs through the system (System Liquid) is free of particulate matter and thoroughly degassed.

11. Fill a clean 500 mL flat-bottomed glass jar with 250 mL of filtered Milli-Q grade water.
  ***Note:*** The appropriate bottle should have a neck with a 41 mm diameter with a 21 mm height to ensure the appropriate stopper (GL45 see below Step 2) fit.
  ***Note:*** The flat-bottomed glass jar must have been washed with Milli-Q water 2-3 times to ensure no salts are left after drying.
  ***Note:*** Only use nitrocellulose based 0.22 μm filters for this and not nylon to avoid shedding polymers.
  a. Swirl filtrate to ensure no visible particulates are detectable. If so, pass filtrate through the filter again.
    **CRITICAL:** Any visible particulates left in the system liquid will clog the PDC and affect uniform droplet deposition during SkinCom construction.
  b. Heat the filtered liquid in a microwave with the cap loose for 2–3 min at full power to release dissolved gases but stop intermittently to ensure the liquid does not boil over.
    ***Note:*** Microwaves with “intermittent heating” settings can also be useful for this step.
  c. Swirl the hot glass bottle in between with the cap lose to release all dissolved bubbles (should originate from the bottom of the bottle and rise to the top).
    ***Note:*** Use thermal hand and eye protection when handling the glass bottle.
12. Once all bubbles have been released, plunge the bottle into a bucket filled with ice and replace the cap with a butyl rubber stopper (Cat# GL45).

**Pause Point:** Wait for system liquid to cool to 18–22^o^C (∼15 min).

13. Transfer the prepped system liquid to the CellenONE X1 system liquid holder (Figure 1A).

**Figure 1:**
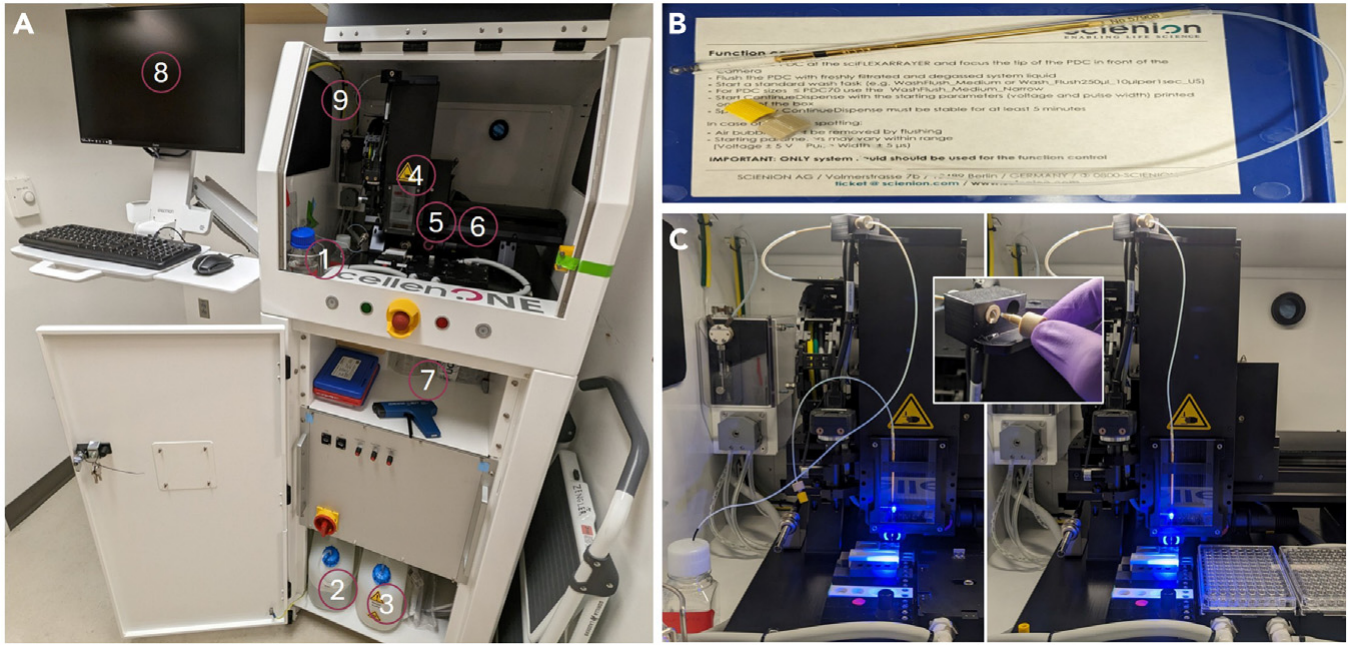
Setup of the CellenONE-X1 for construction of reproducible synthetic microbial communities (A) The full set up of the CellenONE-X1 with the following labeled parts: 1) degassed and filtered system liquid in the system liquid holder, 2) Wash bottle, 3) Waste bottle, 4) Dispense head without PDC, 5) Target location, 6) Probe location, 7) Zerostat 3 MILTY Anti-Static gun, 8) computer interface. (B) A PDC safely stored in the PDC box with the glass nozzle protected. (C) (Left) attached dispense head with the PDC prior to flushing, (inset) appropriate way to connect PDC tubing to the manifold, (right) fully primed system with PDC docked at the camera station.
14. Replace the butyl rubber stopper with the CellenONE X1 printer’s bored cap with capillary tubing.

**CRITICAL:** Ensure the capillary tubing’s end is beneath the system-liquid’s meniscus to prevent clogging the system with air bubbles.

**Pause Point:** The system liquid can be used for droplet generation any time over the next 24 h. Beyond that, the liquid needs to be remade to ensure the PDC is supplied with sufficiently degassed system liquid.

15. The Wash bottle is located in the bottom shelf of the CellenONE X1 (Figure 1A). Fill this bottle up to ½ of capacity (∼1 L) with MilliQ water.

**CRITICAL:** Ensure the bottle does not run dry during the CellenONE X1 operation.

**CRITICAL:** Ensure the filter-protected tubing end is submerged completely in the wash bottle liquid.

16. The Waste bottle is located in the bottom shelf of the CellenONE X1 (Figure 1A).

**CRITICAL:** Ensure the waste bottle has been emptied before starting the run. Add a splash (∼10 mL) of bleach to decontaminate the incoming waste stream.

### Priming the system and validating droplet integrity

**Timing: ∼2 h**

The generated droplet from the CellenONE can be adjusted for shape and size using the piezoacoustic controls. The parameters should be fixed prior to beginning community construction.

17. Retrieve the PDC from the capillary box and carefully remove the nozzle protector (Figure 1B).
  a. Mount the PDC into the dispenser head that is magnetically attached to the face of the arm and replace the head against the arm.
  b. Leave the connector at the end of the PDC disconnected for now (Figure 1C).

**CRITICAL:** The PDC is an extremely fragile component, with the tip of the nozzle made of very thin glass. Users must take much caution to not break or crack the nozzle head while manipulating the PDC.

18. Start the CellenONE X1 robot. Open the software (v.1.89p_USER) and hit “Init Axis”. Allow the robot arm and peristaltic pump initialization routine to complete.

***Note:*** The arm will move forward and sideways to reach the 3D coordinate (x,y,z; 0,0,0) . Ensure nothing is in the way of the arm.

***Note:*** Operate the software in “Simulation” mode for non-robot interface.

**Figure 2:**
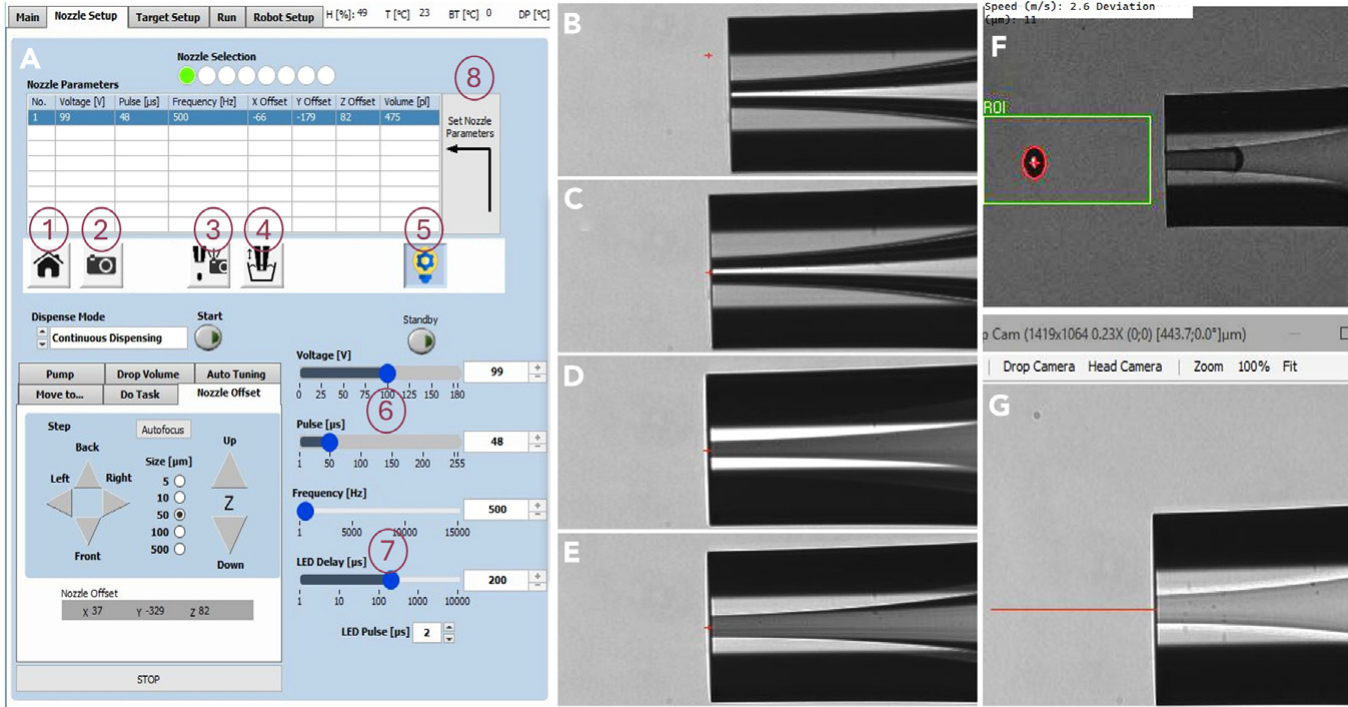
Setting the PDC nozzle at the focus point and adjusting parameters to obtain optimal droplet shape and size (A) Nozzle setup menu on the software to set PDC camera station location using the following buttons: (1) Home station which brings the PDC to safe location away from any obstructions, (2) Camera station which brings the PDC to in front of the camera. This position can be adjusted using the Nozzle offset subtab using the Step buttons, (3) Auto-drop-detect button to create a single drop at the camera station and take a snapshot, (4) Nozzle wash which dips the PDC nozzle into a stream of fresh Milli Q water to clean the outside of the nozzle, (5) Light switch to illuminate the nozzle in front of the camera, (6) Voltage and pulse parameters used to adjust the droplet shape and speed when dispensed from the nozzle, (7) Frequency and LED delay to affect droplet image quality from camera and can be left at their default values, (8) Set Nozzle Parameters button to save the location of the PDC nozzle in front of the camera. (B–E) PDC nozzle as seen by the camera when the nozzle is (B) off center, (C) centered but off-focus, away from the camera, (D) centered but off-focus towards the camera, (E) centered and focused. (F) Optimal, fast-droplet shape and speed when dispensed from the nozzle. (G) Distance of optimal fast droplet from the nozzle.

19. In the software, under the Main tab, hit “Prime” and follow wizard instructions.
  a. Follow the wizard instructions and attach the provided flush bottle to flush the PDC (Figure 1C).
    ***Note:*** When instructed to connect the flush bottle into the manifold nut, only finger tighten to ensure the thin tubing does not get crushed and introduce pressure fluctuations through the tubing.
  b. After flushing the PDC, detach it following wizard instructions and hit “OK”.
  c. Once a droplet of water appears in the connection manifold, connect the PDC tubing end into this manifold, piercing the droplet of water as you tighten (finger tighten only) (Figure 1C inset). This ensures no air bubbles are introduced into the system.
    **CRITICAL:** Connecting the PDC to manifold prior to appearance of droplet will create a large air gap that can damage the capillaries in the PDC. The droplet may take time to appear, be patient.
20. After priming, adjust the droplet size and shape, and the coordinates for the camera station location using commands prompted by the wizard under the Nozzle setup tab.
21. In the software, under Nozzle Set up tab → Nozzle offset sub tab, move the PDC to the camera position by selecting the “camera” button to prepare for positioning (Figure 2A).
  a. The nozzle head as seen by the camera is now visible on the software.
  b. Using arrow keys adjust the nozzle head such that the ejection point of the nozzle head intersects over the center of the camera field of view (green “+” sign) (Figures 2B and 2C). Preset the step sizes to 5 or 10 μm to achieve precise positioning.
  c. The y coordinate of the nozzle head, which dictates the distance of the nozzle head from the camera, needs to match the focus of the camera objective.

**CRITICAL:** To minimize false detections, ensure the nozzle is as close to the focus as possible using the nozzle outlines on the camera live-feed (Figure 2E). If the lines are faint and blurred, the nozzle is off focus away from the camera (Figure 2C). Take small (5 μm) steps towards the camera. If the lines are exaggerated and dark, the nozzle is off focus towards the camera (Figure 2D). Take small (5 μm) steps away from the camera.

22. Under the Nozzle Set up tab, hit the “Continuous dispense” button, setting it to 10–100 droplets (Figure 2A).
  a. Observe the shape of the droplet and the distance of the droplet from the nozzle head. The droplet needs to be circular, stable and along the horizontal of the nozzle head with minimum deviations to its fly-path (Figures 2F and 2G).
23. Use the “voltage”, “pulse” and “frequency” parameters to adjust droplet shape and size.
  a. The voltage and pulse are initially set to the PDC box recommended settings and verify the droplet size and shape by hitting the auto-drop dispense once.
  b. Since we are dispensing microbes in our drops (much smaller and lighter than eukaryotic cells/mammalian cells), the droplet size needs to be as small as possible and its speed needs to be as high as possible without compromising droplet structure and stability.
  c. To achieve this, increase the voltage and pulse such that the droplet is as far away from the nozzle head as possible at the time of camera capture. A distance of 550–600 μm from the nozzle head is optimal.

***Note:*** Specs suggested for SkinCom construction are Voltage: 91 V and Pulse of 52 μs. This should create a droplet with a speed greater than 1.5 m/s at a distance of ∼450 μm from the nozzle head.

***Note:*** The distance of the droplet from the nozzle head can be measured by using the annotation tool in the image window and drawing a straight line from the nozzle to the center of the droplet.

24. Once satisfied with the positions, the shape and distance from nozzle of the droplet generated, hit “SET NOZZLE PARAMETERS” to ensure the camera station position of the nozzle and parameters is saved for the duration of the session (Figure 2A).

## Key resources table

**Table:** 

REAGENT or RESOURCE	SOURCE	IDENTIFIER
**Chemicals, peptides, and recombinant proteins**		

L-cysteine	Sigma-Aldrich	Cat# 168149
Hemin and vitamin K	VWR	Cat# 75803-006
Triton X-100	Sigma-Aldrich	Cat# X100-100ML
Sodium lauryl sulfate (SLS)	https://corporate.evonik.com/en	SLS
Sodium lauryl ether sulfate (SLES)	https://corporate.evonik.com/en	SLES
Rhamnolipid (RL)	https://corporate.evonik.com/en	RL
Creatine	https://corporate.evonik.com/en	Creatine

**Critical commercial assays**		

ZymoBIOMICS DNA/RNA miniprep kit	Zymo Research	Cat# R2002
QIAGEN DNeasy PowerSoil Pro kit	QIAGEN	Cat# 47016
Qubit dsDNA, high sensitivity	Thermo Fisher Scientific	Cat# Q32851
Nextera XT DNA library kit	Illumina	Cat# FC-131-1096 and FC-131-2001

**Deposited data**		

Sequences for *in vitro* experiment - assembly	This paper	SRA: SUB14340232
Sequences for *in vitro* experiment - compounds	This paper	SRA: SUB14340534
Sequences for *in vivo* experiment - mouse	This paper	SRA: SUB14342487
Sequences for *in vivo* experiment - human	This paper	SRA: SUB14347828

**Experimental models: Organisms/strains**		

*Corynebacterium afermentans*: strain background: ATCC 51403, strain CIP 103499 (LCDC 88199)	ATCC	ATCC: 51403, strain CIP 103499 [LCDC 88199]
*Cutibacterium acnes*: strain background: ATCC KPA171202	ATCC	ATCC: KPA171202
*Micrococcus luteus*: strain background: ATCC 4698	ATCC	ATCC: 4698
*Staphylococcus aureus*: strain background: ATCC 35556, strain SA113	ATCC	ATCC: 35556, strain SA113
*Staphylococcus capitis*: strain background: ATCC 27840, strain LK 499	ATCC	ATCC: 27840, strain LK 499
*Staphylococcus epidermidis*: strain background: ATCC 12228	ATCC	ATCC: 12228
*Staphylococcus hominis*: strain background: ATCC 27844, strain DM 122	ATCC	ATCC: 27844, strain DM 122
*Staphylococcus warneri*: strain background: ATCC 27836, strain AW 25	ATCC	ATCC: 27836, strain AW 25
CD-1 mice, strain 022, sex: M, age: 8 weeks	Charles River Laboratories	Strain code: 022

**Software and algorithms**		

Trimgalore	The Babraham Institute^6^	https://github.com/FelixKrueger/TrimGalore
bowtie2 (v.2.2.3)	Langmead and Salzberg^7^	https://github.com/BenLangmead/bowtie2
phyloseq	MCMurdie and Holmes^8^	https://joey711.github.io/phyloseq/
Growthcurver	Sprouffske and Wagner^9^	https://cran.r-project.org/web/packages/growthcurver/vignettes/Growthcurver-vignette.html
Vegan 2.6-10	Oksanen et al.^10^	https://cran.r-project.org/web/packages/vegan/index.html

**Other**		

Codes used to generate figures in this manuscript	This manuscript	https://zenodo.org/records/14908419
Brain heart infusion (BHI)	MilliporeSigma	Cat# 53286
Depilatory cream	Nair	http://www.naircare.com/en/products
Transparent film dressing (3M Tegaderm)	3M	Cat# 1626
Sterile cotton swabs	Fisher	Cat# 22-029-630

## Step-by-step method details

### Characterizing growth rates of SkinCom community bacteria

**Timing: 7 days (varies by microbe)**

This step is necessary for obtaining accurate growth characteristics of the constituent members of the SkinCom that will aid with constructing the synthetic community with controlled abundance levels of constituent members.

1. Revive bacteria from glycerol stocks of purchased strains of the 9 SkinCom members in 1x Brain Heart Infusion (BHI) medium and incubate at 37°C.

***Note:*** All strains selected for this synthetic community can grow on 1X BHI, however, if alternate strains are chosen by the user, appropriate media can be used instead.

***Note:****Cutibacterium acnes* needs to be revived in degassed and reduced (4 mM cysteine) BHI in the anaerobic chamber with an atmosphere of N2/CO2/H2 (85%/10%/5%) or using stoppered tubes following the Hungate technique.^11^

2. After visible growth in all tubes (either cloudiness in media or an Optical Density at 600 nm (OD 600) > 0.3 after subtracting for background), streak the cultures on BHI-agar (1.5% w/v) plate to check for purity and incubate at 37°C.

***Note:****Cutibacterium acnes* needs to be streaked onto a degassed BHI plate in the anaerobic chamber with an atmosphere of N2/CO2/H2 (85%/10%/5%).

3. After colony formation, pick a single colony for each strain and revive them in fresh 1x BHI media and incubate at 37°C.

***Note:****Cutibacterium acnes* needs to be revived in degassed BHI in the anaerobic chamber with an atmosphere of N2/CO2/H2 (85%/10%/5%) or using Hungate tubes.^11^

4. After visible growth in all tubes, dilute the cultures in fresh 1x BHI to a final optical density at 600 nm wavelength (OD600) of 0.07.
5. Transfer 200 μL of the OD600 normalized cultures into predetermined locations in a 384-well plate.
  a. This 384-well plate represents the probe plate for the CellenONE X1 and should be placed into the probe location of the printer (Figure 1A).

***Note:*** Alternatively, a 96-well plate can be used as a probe for the CellenONE X1.

6. For the fresh plate into which fixed volumes of the pre-cultures will be dispensed (target plate), fill a new 96-well plate with fresh 1x BHI. The plate is placed in the target location (Figure 1A).
7. In the CellenONE X1 software, under the Main tab, select the appropriate Probe plate (“MTP384”) and Target plate (“MTP96”).

***Note:*** Custom targets and probes such as microscopic slides, petri dishes, etc. can also be used. In the robot setup, define specific 3-dimensional coordinates for the individual spot points (for the target destinations/wells) and acquisition points (for the probe plate origins/wells), and instruct the arm to dispense into and aspirate from these locations. For more details, contact manufacturers.

**Figure 3:**
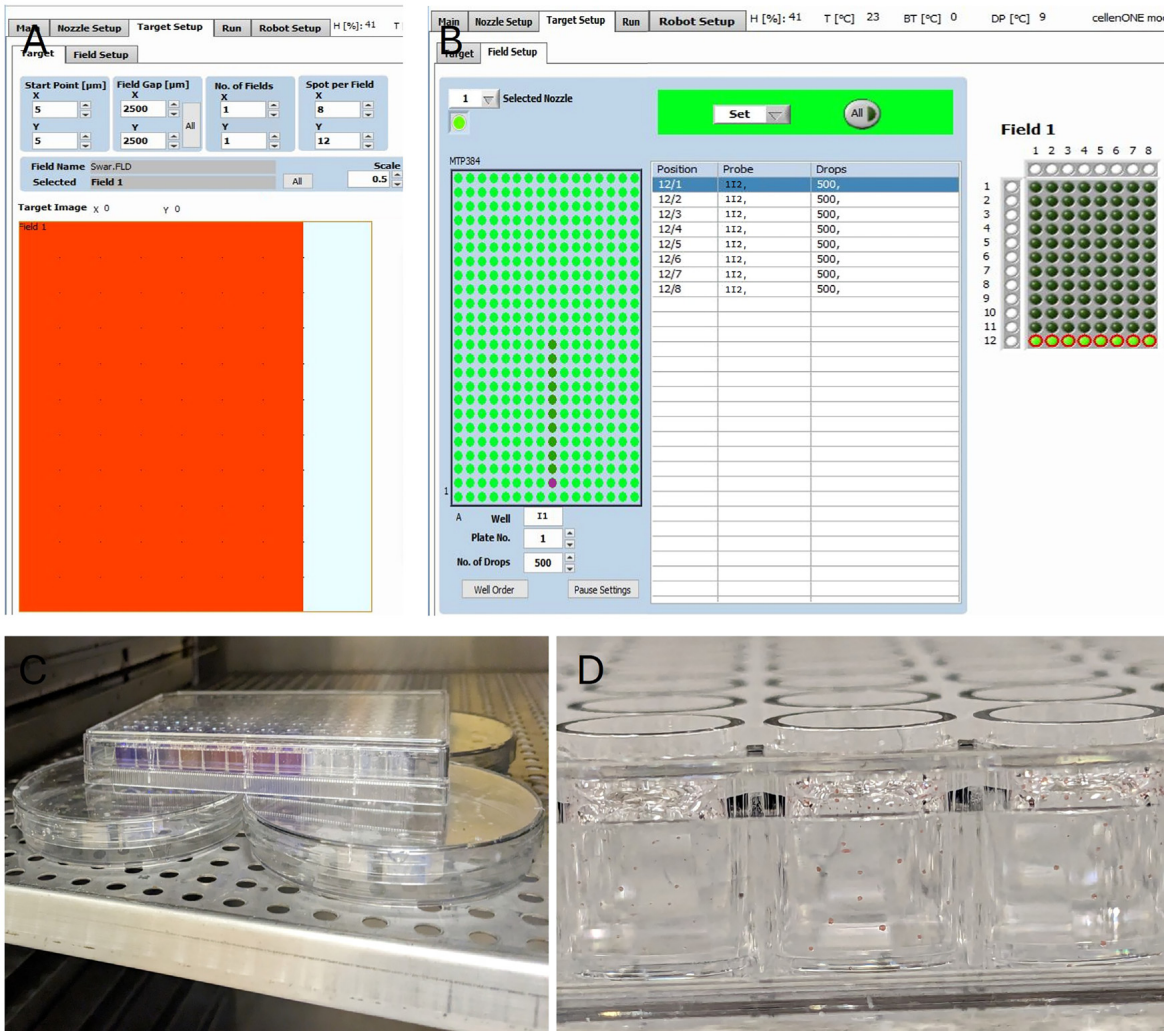
CellenONE X1 software for target and field setup (A) Target map layout for a MTP96 well plate target displayed on an x and y axis coordinate plane. The spots in the field indicate the individual wells. The spot positions can be adjusted to center the nozzle over each well when dispensing using the robot setup tab. (B) Field map of the probe and target plates to configure the probe-well to target-well map and the number of droplets to be dispensed. Here, the field map reflects each well in the 12^th^ column of the target 96 well plate and will receive 500 drops of the contents in probe plate well I1. (C) Incubate the prepped target plate at 37°C on top of 4 petri-dishes filled with water to create a local humidified environment that minimizes evaporative loss. (D) Target well after cellenone_AutoDrop run completes, visualized by using droplets of food-dye dispensed into mineral oil.
9. Go to the Target Setup tab → Field Setup sub tab. Verify the probe plate matches the MTP384 probe configuration by noting 384 acquisition points/wells for the probe plate on the left of the screen. The configuration of the Target plate should be clearly visible on the right of the screen (Figure 3B).
10. Manually select the acquisition well in the probe plate for each OD600 normalized bacterial culture. Specify the number of droplets for all as 200. Then manually select the destination wells for each strain in the target plate.

***Note:*** The choice of droplet numbers for each strain is arbitrary and dependent on the user. 200 was chosen to ensure all strains started with a similar number of cells that are at mid to late exponential phase.

***Note:*** When designing your target plate layout, add a one row gap between different species of bacteria to prevent cross contamination. Also leave the surrounding wells as controls to avoid edge effects on the data.^12^

***Note:*** Double check the manual assignments of the destination wells for each bacterium to ensure there is no duplicate assignment of target wells leading to contamination.

8. Next, under the Target Setup tab → Target sub tab, verify that the target image matches the MTP96 target configuration by ensuring that there are 8 spots along the X axis field and 12 spots axis along the Y field, indicating the 96 destinations/wells in the plate (Figure 3A).

11. After confirming the target and field layouts, save the field. This field will be auto loaded at run unless a different field is loaded.
12. Switch to the Run tab. Ensure the correct Run “cellenone_AutoDrop” was selected in the Main tab. Refer to Methods video S1 for a full description of all the steps executed as part of “cellenone_AutoDrop”. This pre-loaded run mode makes the PDC sequentially perform the following:
  a. Aspirate 10 μL of sample from each probe plate acquisition well.
  b. Check for a single, unbroken drop created at the camera station using the settings entered during system priming.
  c. Dispense the specified number of droplets (200 here) into the corresponding target plate destination well.
  d. Re-check the droplet integrity at the camera station.
  e. Wash the PDC at the washing station to prepare for the next probe plate acquisition well.

**CRITICAL:** Before starting the run, ensure the workspace in front of the robot arm is completely clear of objects, equipment or any other obstacles. Keep clear of the robotic arm when in motion. The arm moves fast, and the exposed nozzle is extremely delicate.

13. After the run completes, cover the plate using the anti-condensation coated plate lid and incubate the plate at 37°C.
  a. Create a local humidified environment around the plate in the incubator by placing the plate on 4 petri-dishes filled with water to minimize evaporative loss (Figure 3C).
  b. Take regular readings of the plate wells using a spectrophotometer for the next six days.

Methods video S1. Full sequence of the cellenONE X1 pico-printer followed for cellenone_AutoDrop command, related to step-by-step-method details, step 11

### Estimating SkinCom proportions using growth curve metrics

**Timing: ∼2 h**

Estimating the adjusted proportions to combine each bacteria ensures the constructed community has all strains growing together rather than being dominated by a single, fast-growing strain. This step details the calculation and construction of multiple iterations of the community. The final model community, designated SkinCom, was chosen based on alpha diversity and representativeness to native human skin.^1^

14. Use the growthcurver package^9^ to analyze the OD600 data from all the incubated plates to calculate the growth rate and the time to mid-log phase for all the members of the community.

***Note:*** The growth metrics can also be manually computed using Excel or other sheet software, computing the slope as the growth rate and the time to mid-exponential phase for each curve.

15. Calculate the proportions by which to combine community members, based on grouping them by their growth rates (2 groups (2x cutoff) and 3 groups (3x cutoff) combinations), or by normalizing to the strain fastest to grow or fastest to reach mid log phase (Growth Curve Slope (GCS) adjusted and Growth Curve Time (GCT) adjusted combinations), or by mixing them equally (Equal Mixed (EM)) (Table 1).

**Table 1:** Droplet-based proportions for constructing growth rate adjusted (2x and 3x), fastest to log phase adjusted (GCS), time to log phase adjusted (GCT), or no adjusted (EM) communities

Organism	2x cutoff	3x cutoff	GCS	GCT	EM
*Corynebacterium* afermentans (ATCC 51403, strain CIP 103499 [LCDC 88199])	2,000	200	835	10,000	200
*Cutibacterium acnes* (ATCC KPA171202)	2,000	200	2,000	10,000	200
*Micrococcus luteus* (ATCC 4698)	2,000	2,000	510	304	200
*Staphylococcus aureus* (ATCC 35556, strain SA113)	2	2	83	3	200
*Staphylococcus capitis* (ATCC 27840, strain LK 499)	2	2	161	27	200
*Staphylococcus epidermidis* (ATCC 12228)	2	2	242	7	200
*Staphylococcus hominis* (ATCC 27844, strain DM 122)	2	2	178	9	200
*Staphylococcus warneri* (ATCC 27836, strain AW 25)	2	2	54	6	200
*Streptococcus mitis* (ATCC 49456, strain NCTC 12261*)*	2,000	200	1,472	319	200
Media only (1X or 0.1X BHI)	0	0	0	0	0
**Total droplets**	**8,010**	**2,610**	**5,535**	**20,673**	**1,800**
**Total volume of inoculum (μL)** (avg droplet volume = 350 pL)	**2.80**	**0.91**	**1.94**	**7.24**	**0.63**

### Community assembly using CellenONE X1

**Timing: 6 h assembly, 3 days incubation**

Various iterations of the community can be constructed by combining the members based on proportions computed from adjusted growth rates or normalized to fastest growers. The ideal community based on reproducibility, alpha diversity and representativeness when compared to human skin microbiome data can be selected following this.

16. Construct the community from fresh pre-cultures of bacterial strains normalized to an OD600 of 0.07 as described previously (step-by-step method details, steps 1-4) by combining the strains at the calculated proportions from Table 1.

***Note:*** Reproducibility and stability of the community must be verified by at least 4 replicates.

17. Set up and prime the CellenONE X1 as described in the priming the system and validating droplet integrity section of the protocol.
  a. Fill the probe plate with the bacteria normalized to OD600 of 0.07.
  b. Fill the target plate with 200 μL of 1X BHI.
  c. Insert the probe and target plates in their appropriate locations.
18. Load the community construction field file (provided in Supplementary Data) and follow Setup Target steps as outlined earlier (step-by-step method details, steps 8 - 10 ).
19. After the community construction run completes, cover the plate using the anti-condensation coated plate lid and incubate the plate at 37°C.
  a. Create a local humidified environment around the plate in the incubator by stacking the plate on 4 petri-dishes filled with water to minimize evaporative loss.
  b. Take regular readings of the plate wells using a spectrophotometer for the next 96 h.

### DNA extraction from constructed communities

**Timing: ∼variable (depends on extraction protocols and capabilities)**

DNA extraction from all communities constructed is performed using the Qiagen DNeasy PowerSoil Pro Kit, following the manufacturer’s instructions using community pellets. The protocol involves 5 key steps - lysis (chemical and mechanical), inhibitor removal, DNA binding, washing, and elution.

20. For sample lysis, transfer the contents from each well in the target plate to a fresh Eppendorf tube (1.5 mL).
21. Spin down the samples at 10,000 g for 2 min and discard the supernatant.

***Note:*** Include transfer of the content from the negative control wells to account for contaminating microbial DNA during sample prep.^13^

22. Resuspend the pellet in 800 μL of CD1 solution provided in the kit. Pipette mix briefly or vortex.
23. Transfer the entire contents to a PowerBead Pro tube provided in the kit.
24. Perform mechanical lysis in tandem with chemical lysis (CD1 solution) by securing the PowerBead pro tubes to a Vortex Adapter horizontally. Vortex at max speed for 12 min.
25. Follow the manufacturer’s instructions until pure, inhibitor free DNA is eluted, which can be verified using Qubit (Invitrogen), NanoDrop (Thermo Scientific), or even a BioAnalyzer (Tape Station) (Agilent).

***Note:*** DNA can be eluted using RNAse-free water instead of C6 solution.

26. Quantify the concentration of DNA extracted using the Qubit HS DNA assay.

***Note:*** Ensure the Qubit has been calibrated using manufacturer provided standards.

**Pause Point:** DNA can be stored at −20 C indefinitely after this step, however, avoid frequent freeze-thawing cycles.

### Library preparation for shotgun sequencing of constructed communities

**Timing: ∼variable (depends on sequencing turnaround)**

DNA from microbial communities collected from all samples are prepared for sequencing using Illlumina’s Nextera-XT DNA library preparation kit following the manufacturer’s protocol. The protocol involves 4 key steps: tagmentation of DNA, index-PCR and library amplification, post-amplification cleanup, and quantification.

27. For all samples, transfer 5 μL of 0.5 ng/μL normalized extracted DNA as input for tagmentation.
28. Follow manufacturer’s instruction for addition of appropriate amounts of Amplicon Tagment Mix (ATM) and Tagment DNA Buffer (TD).

***Note:*** Also input 5 μL of extracted controls to account for reagent and laboratory contamination.^13^

***Note:*** Optionally sequence a positive control like ZymoBIOMICS Microbial Community Standard to identify and eliminate amplification bias.

29. Incubate the mix in a thermal cycler at 55°C for 10 min to complete the tagmentation.
30. Bring the sample back to 10°C and add the manufacturer-specified amount of Neutralize Tagment Buffer (NT) to stop the tagmentation.

**CRITICAL:** Add the NT buffer as soon as the sample temperature returns to 10°C to completely inactivate the transposome and avoid over-tagmenting.

31. Use 10 μL of Nextera-XT Index kit v.2 indices for indexing libraries and amplification.

***Note:*** Use dual index barcoding to increase multiplicity of samples sequenced in the same run. Note down the index combinations used.

**CRITICAL:** Ensure each sample has a unique index or combination of indices.

32. Amplify samples for 12 cycles following the amplification protocol specified by the manufacturer.

***Note:*** One can also use only 9 μL of index and spike in 1 μL of 1x SYBR Green mix into each sample to follow amplification of libraries if using a qPCR machine. Delayed cycle qt can indicate quality of tagmentation and extracted DNA.

33. Libraries are cleaned after amplification following manufacturer’s instructions.
34. Quantify libraries using Qubit HS DNA assay.

***Note:*** Ensure the Qubit has been calibrated using manufacturer provided standards.

35. Normalize each of the libraries to 10 nM concentration.
36. Pool the normalized libraries together, by combining at most 150 samples per lane to ensure at least 5 million reads per sample when sequencing on a NovaSeq 6000 (see next step).

**Pause Point:** Pooled libraries can be stored at −20°C indefinitely.

37. Send libraries to sequencing core/company for paired-end 150 sequencing on NovaSeq 6000.
  a. Sequence at least 5 million reads per sample. Request 5% PhiX spike-in.

### Data preprocessing and analysis to identify ideal community (SkinCom)

**Timing: ∼variable (depends on computational power)**

The analysis of the sequenced data from all samples will indicate which community construction proportions resulted in an assembly (across replicates) that has the highest alpha-diversity (using the Shannon-diversity metric) and is most representative of human skin (compared with public skin microbiome datasets). Recommended computation requirements include 130 GB memory running on Intel Xeon CPU E5-2620 processor with an E7 bridge. All steps listed here also support multithreading, which can be achieved with these hardware features.

The preprocessing includes 4 main steps: quality check and trimming, indexing the skin microbe members, aligning, and constructing a feature-table.

38. Install required packages with Conda (Anaconda Software Distribution. Computer software. Version 2-2.4.0. Anaconda, Nov. 2016. Web).
  ***Note:*** It is recommended to install all required packages using Conda, an open-source package management system. The specific installation instructions for each operating system can be found in the Conda documentation. This protocol describes downloading Miniconda, which is a miniature installation of Anaconda.
  a. Make a directory for conda and download the installation script.
    >mkdir ∼/miniconda3
    >wget https://repo.anaconda.com/miniconda/Miniconda3-latest-Linux-x86_64.sh -O ∼/miniconda3/miniconda.sh
  b. Run Installation.
    >bash ∼/miniconda3/miniconda.sh -b -u -p ∼/miniconda3
    ***Note:*** Optionally cleanup by running:
    >rm ∼/miniconda3/miniconda.sh
39. Create a Conda environment and install all required packages:

>conda create -n metagenomics_pipeline -c bioconda trim-galore bowtie2 woltka python=3.8

40. Activate the Conda environment before processing metagenomic reads.

>conda activate metagenomics_pipeline

41. Index the SkinCom genomes by using the reference sequences downloaded from NCBI earlier (Table 2).

**Table 2:** SkinCom strain names, phylogeny, and NCBI nucleotide and taxonomy IDs

Strain name	ATCC strain	NCBI nucleotide	Genus	Species	NCBI taxonomy ID
*Corynebacterium afermentans* subsp. afermentans	ATCC 51403, strain CIP 103499 [LCDC 88199]	NZ_LXGG01000001	*Corynebacterium*	*afermentans*	144183
*Cutibacterium acnes* (ATCC KPA171202)	ATCC KPA171202	NC_006085.1	*Cutibacterium*	*acnes*	267747
*Micrococcus luteus* NCTC 2665	ATCC 4698	NC_012803.1	*Micrococcus*	*luteus*	465515
*Staphylococcus aureus* subsp. aureus SA113	ATCC 35556, strain SA113	NC_007795.1	*Staphylococcus*	*aureus*	93061
*Staphylococcus capitis* subsp. capitis	ATCC 27840, strain LK 499	NZ_CP007601.1 NZ_CP007602.1	*Staphylococcus*	*capitis*	72758
*Staphylococcus epidermidis* ATCC 12228	ATCC 12228	NC_004461.1 NC_005008.1 NC_005007.1 NC_005006.1 NC_005005.1 NC_005004.1 NC_005003.1	*Staphylococcus*	*epidermidis*	176280
*Staphylococcus hominis* subsp. hominis	ATCC 27844, strain DM 122	NZ_CP033732.1 NZ_CP033731.1 NZ_CP033733.1	*Staphylococcus*	*hominis*	145391
*Staphylococcus warneri* AS 125	ATCC 27836, strain AW 25	NZ_LR134269.1	*Staphylococcus*	*warneri*	1292
*Streptococcus mitis* NCTC 12261	ATCC 49456, strain NCTC 12261	NZ_CP028414.1	*Streptococcus*	*mitis*	28037

>bowtie2-build <reference_genomes.fasta> <output_path_and_index_prefix> --threads <number_of_threads>

***Note:*** The reference genomes should be in the form of a single fasta file, with each header representing one SkinCom species.

***Note:*** One example of <output_path_and_index_prefix> can be ./skincom_index/skincom which generates bowtie2 index files in the directory of ./skincom_index/ and all files should have the prefix skincom (e.g.skincom.1.bt2).

42. Proceed with preprocessing the metagenomic reads for each of the constructed communities submitted for sequencing along with the extraction controls.
  a. First, apply adapter and quality trimming to raw paired-end metagenomic reads using Trim Galore.
    > trim_galore --paired <metagenomic_R1_filename> <metagenomic_R2_filename> --quality 20 --fastqc -o <output folder>
  b. Concatenate trimmed files from R1 and R2 of the same sample.
    >cat <sample_name>∗R1∗trimmed∗.fq.gz <sample_name>∗R2∗trimmed∗.fq.gz > <sample_name>_trimmed.fq.gz
  c. Create directories to store alignment results. Here is an example of creating directories under the home directory.
    >mkdir -p ∼/aligned/samfiles; mkdir -p ∼/aligned/bowfiles
  d. Align trimmed reads to SkinCom reference genomes using Bowtie2.
    >bowtie2 -U <sample_name>_trimmed.fq.gz -x <output_path_and_index_prefix> -p <number_of_threads> -S ∼/aligned/samfiles/<sample_name>.sam 2> ∼/aligned/bowfiles/<sample_name>.bow
    ***Note:*** Sequence alignment and map (SAM) files represent the alignment information whereas .bow files provide an overview of alignment statistics such as number of reads processed, number of reads aligned, and overall alignment rate.
    ***Note:*** If storage is a concern, consider adding --no-header and --no-unal to reduce the size of the generated SAM files by omitting headers and unaligned reads, respectively.
  e. Generate a taxon count table using the following command. The resulting tab-separated values (TSV) file lists the counts of each strain across all samples.
    >woltka classify -i ∼/aligned/samfiles -o ∼/taxons.tsv
43. Deactivate Conda environment.

>conda deactivate

### Applying SkinCom to a mouse model for reproducible, multiomic study

**Timing: ∼1 week**

The reproducible SkinCom constructed following the ratios computed and verified using the CellenONE X1 can now be used as part of model systems to study the effect of chemical and/or environmental factors on the skin microbiome. In this use-case, the growth curve slope adjusted community in 1x BHI (GCS_1x BHI) is used in a mouse model at varying levels of dilution to test the effect of biomass on the skin microbiome. Animal experiments were conducted in accordance with the rules and regulations of the Institutional Animal Care and Use Committee, which was approved by the UC. San Diego IRB protocol S00227M.

44. Obtain 8-week-old CD1 mice from the vendor (Charles River Laboratories, n = 5 mice/group).
45. House mice in filter-top cages with regulated environmental conditions (20°C–22°C, 30–70% relative humidity, 12 h light/12 h dark cycle) for at least 2 weeks prior to the start of the experiment.
46. Shave the dorsal skin, followed by the application of a chemical depilatory agent to the treated area (Nair, USA) under isoflurane sedation wipe the shaved area with a wet sterile gauze.^14^^,^^15^
47. Monitor mice for 24 h prior to SkinCom application, for animal health and dressing (gauze) integrity.
48. Follow cultivation of community microorganisms as done earlier (step-by-step method details, steps 1-4).
49. Manually construct the SkinCom from the OD600 normalized cultures following the ratio computed using the CellenONE X1 earlier for the growth-curve-slope adjusted community in 1x BHI (GCS_1x BHI). See Table 3 for volumes.

**Table 3:** Volumes for manual construction of Growth-Curve-Slope adjusted 1x BHI community (GCS_1x BHI)

Organism	Volume to add (μL)
*Corynebacterium afermentans* (ATCC 51403, strain CIP 103499 [LCDC 88199])	835
*Cutibacterium acnes* (ATCC KPA171202)	2,000
*Micrococcus luteus* (ATCC 4698)	510
*Staphylococcus aureus* (ATCC 35556, strain SA113)	83
*Staphylococcus capitis* (ATCC 27840, strain LK 499)	161
*Staphylococcus epidermidis* (ATCC 12228)	242
*Staphylococcus hominis* (ATCC 27844, strain DM 122)	178
*Staphylococcus warneri* (ATCC 27836, strain AW 25)	54
*Streptococcus mitis* (ATCC 49456, strain NCTC 12261)	1,472
**Total volume**	**5,535**

**CRITICAL:** Ensure properly calibrated pipettes are used when dispensing small, yet accurate volumes. Use reverse pipetting for volumes less than 0.2 μL for greater accuracy.

**CRITICAL:** Perform community construction in a sterile chamber like a biosafety cabinet to ensure no cross-contamination.

***Note:*** The community can also be constructed again using the CellenONE X1 pico-printer as described earlier (step-by-step method details, steps 6 - 13).

50. Construct the dilutions of the community to test the effect of biomass on the skin microbiome using the mouse model.
  a. 106 CFU/mL (high bacterial load).
  b. Add 100 μL of SkinCom master mix to 9,900 μL of BHI to achieve 104 CFU/mL (medium bacterial load).
  c. Add 100 μL of 104 CFU/mL SkinCom master mix to 9,900 μL of BHI to achieve 102 CFU/mL (low bacterial load).

***Note:*** Due to variability across microbial species in OD600 vs CFU correlations due to cell morphologies, along with variation across spectrophotometers being used and the length of the light path from emitter to receiver,^16^^,^^17^^,^^18^ we tested the correlation between OD600 and CFU for the microbes in this community and an OD600 of 0.05 corresponded to 10^6^ CFU/mL.

51. Pellet 1 mL of each SkinCom master mix at 10,000 rpm for 1 min and remove the supernatant. Resuspend in 50 μL of 1x BHI.
52. Spot the 50 μL concentrated SkinCom onto BHI agar patch (2 cm diameter). Allow to dry at 18–22°C.

***Note:*** Include 5 media spots (negative control/ no bacteria). Place agar patch onto sterile gauze.

**Figure 4:**
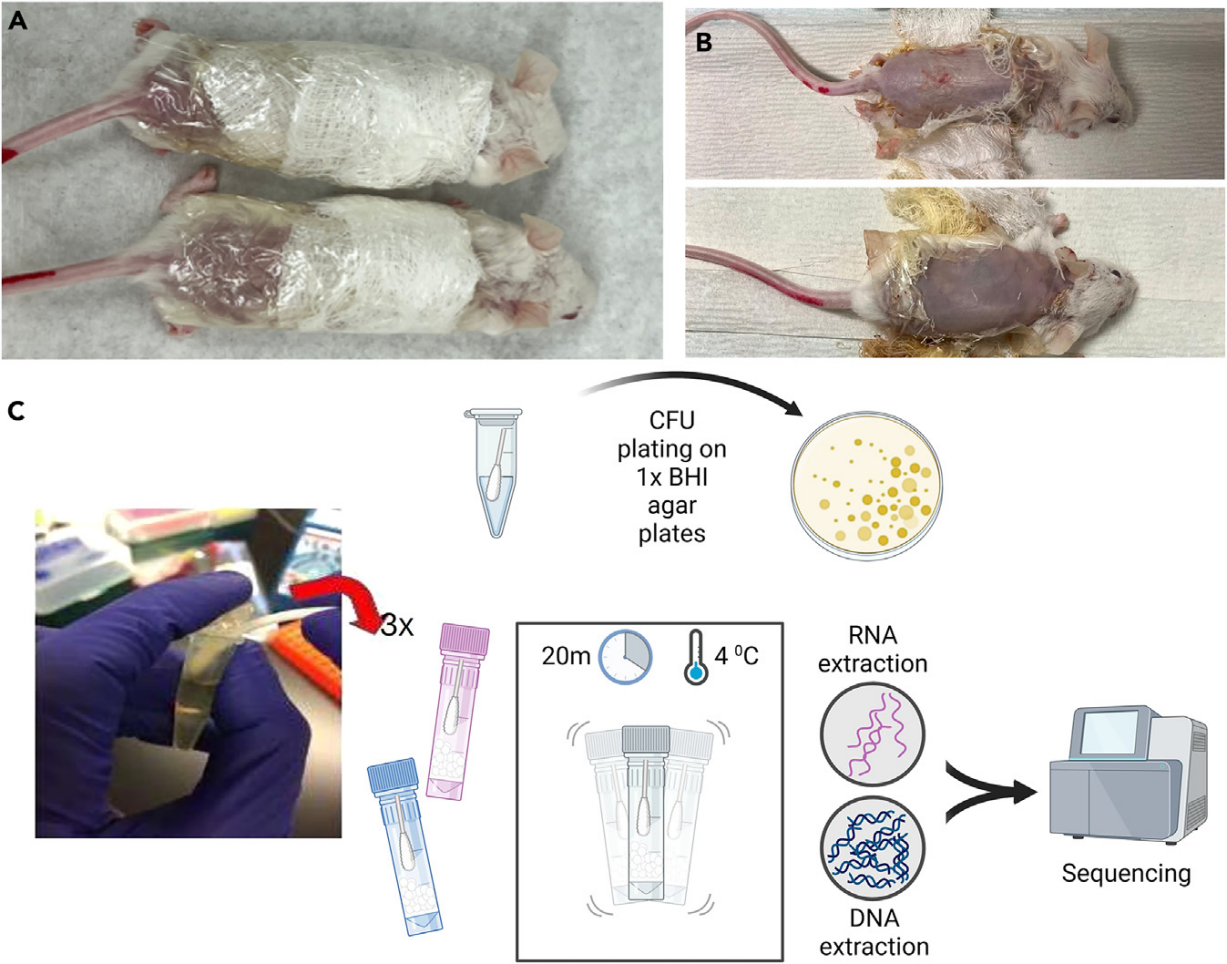
Applying SkinCom to a mouse model for reproducible, multiomic study (A) SkinCom applied to agar patches and fastened securely to prepped mice backs using transparent film dressing. Such fastening ensures the applied community is not removed by the mice for the duration of the experiment. (B) Mice backs are assessed for any lesions prior to swabbing, indicative of inflammation from applied Skincom. (C) Swab tips are snapped off sterilely for CFU counting, DNA and RNA extractions (using bead beating approaches for lysing following ZymoBIOMICS instructions) for metagenomics and metatranscriptomics.
56. Monitor that the application is still intact every 12 h for 3 days.
57. Collect submandibular blood to assess the systemic spread of infection 72 h post SkinCom patch application.

***Note:*** Add 20 μL of heparin per 100–200 μL of blood heparin to prevent blood clots.

53. Place agar patch onto sterile gauze.
54. One cage at a time, sedate the mice under isoflurane.
55. Fasten the gauze containing agar patches onto the shaved skin with transparent film dressing (3M-Tegaderm) (Figure 4A).

58. Perform a serial dilution of the obtained blood. Spot 5 μL onto BHI plates serially diluted on BHI agar plates.

***Note:*** Colony formation on incubated plates after a few days indicate bacterial infection in the blood.

59. Euthanize animals by CO_2_ inhalation.
60. Remove the gauze dressing and make a note of any skin lesion (Figure 4B).
61. Prepare skin swabbing solution, which contains Tris-EDTA (TE) buffer +0.5% Tween 20 + 1% Triton X-100.
62. Submerge three sterile cotton swabs into the skin swab solution.
63. Hold 3 swabs side by side and swab the dorsal skin for 1 min.
  a. Use 1 swab for CFU plating.
  b. Use 1 swab for DNA extraction and metagenomics analysis.
  c. Use 1 swab for RNA extraction and metatranscriptomic analysis.
64. Snap off the tip of one swab for CFU plating in a microcentrifuge tube containing 750 μL of PBS (Figure 4C).
65. Snap off the tips of the remaining two swabs for DNA and RNA extractions in Zymo bashing bead lysis tubes (catalog# S6012-50) containing 750 μL DNA/RNA shield (catalog# R1100-250).
66. Follow the ZymoBIOMICS protocol for DNA and RNA extractions with the following modifications.
  a. Bead-bash cotton swabs for 20 min at 4°C.
  b. During the elution step, add 30 μL of water to the column and incubate at 18–22°C for 5 min before elution.
  c. Repeat this 5-min incubation twice (10 min total).
67. Follow the Nextera XT protocol for metagenomics library prep and sequencing (step-by-step method details, step 27 - 34).
68. Sequence DNA metagenomics samples paired-end 100 on the NovaSeq 6000 platform at ∼10 million reads per sample.
  a. Pool samples accordingly to achieve this sequencing depth per sample.
69. Follow the manufacturer’s protocol for KAPA RNA HyperPrep protocol for metatranscriptomics library prep. Refer to Lekbua et al.^1^ for details.
70. Sequence RNA metatranscriptomics samples paired-end 100 on the NovaSeq 6000 platform at 50 million reads per sample.
  a. Pool samples accordingly to achieve this sequencing depth per sample.

## Expected outcomes

**Figure 5:**
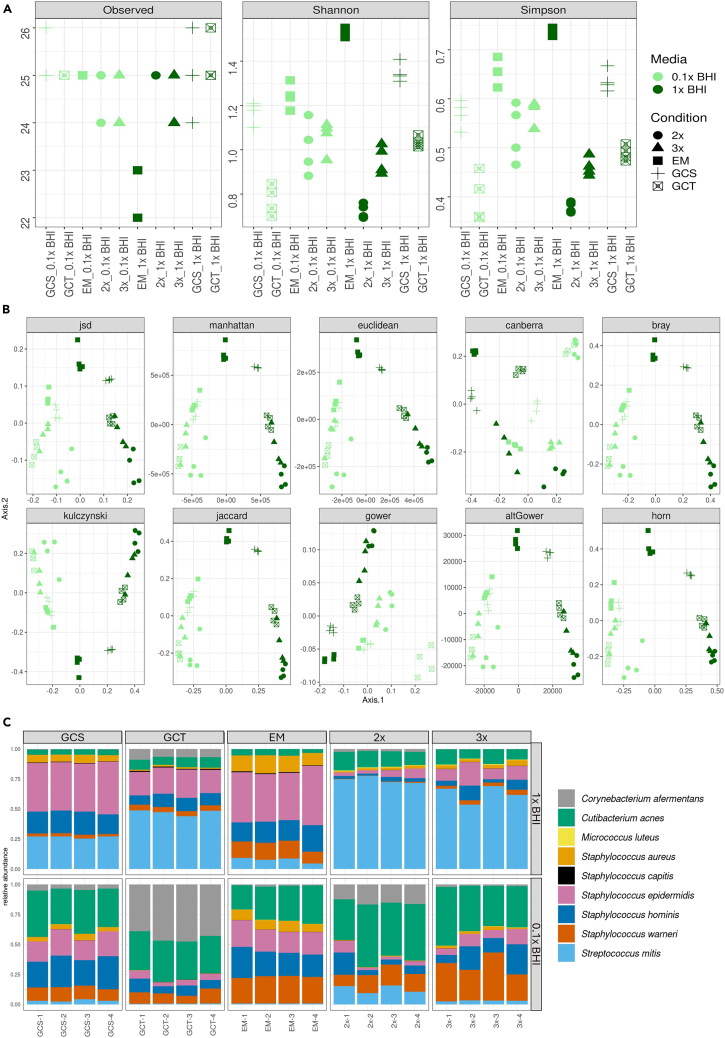
Expected outcomes from experiments using the SkinCom (A) Alpha-diversity metrics of the communities constructed using proportions adjusted for growth rate, fastest to log, time to log or equally mixed, including number of features observed (Observed), richness of features in the samples based on Shannon or Simpson diversity metric. (B) Various distance metrics used to cluster the constructed communities. Communities from GCS 1x BHI and EM 1x BHI almost always cluster with each other. And the variance in the dataset is always explained more by the experiment group (average PERMANOVA F-statistic 229, *p-value* 0.001) than by media (average PERMANOVA F-statistic 47.8, *p-value* 0.001) or by construction ratios (average PERMANOVA F-statistic 4.5 *p-value* 0.004). See full statistics in Table 4. (C) Relative abundance of the 9 member SkinCom across replicates for all adjusted or equally mixed communities in 1x and 0.1x BHI.

SkinCom communities constructed based on the accurate growth rates and metrics adjusted ratios using the CellenONE X1 depict strong reproducibility between replicates, indicative from processed metagenomics fasta files and computed alpha diversity (Figure 5A) and beta-diversity metrics (Figure 5B). The communities cluster stronger by “Experiment” than by “Condition” or “Media” of cultivation based on unweighted or presence/absence distance metrics (see Table 4).

**Table 4:** PERMANOVA computed on the communities based on multiple weighted and unweighted distance metrics

Distance metric	Experiment_ F	Experiment_ Pr(>F)	Media_F	Media Pr(>F)	Condition_F	Condition Pr(>F)
jsd	739.667	0.001	91.563	0.001	5.028	0.003
manhattan	79.779	0.001	44.180	0.001	4.043	0.002
euclidean	94.082	0.001	54.967	0.001	3.017	0.015
canberra	30.823	0.001	9.645	0.001	5.407	0.001
bray	79.779	0.001	44.180	0.001	4.043	0.002
kulczynski	79.779	0.001	44.180	0.001	4.043	0.001
jaccard	41.078	0.001	30.682	0.001	3.917	0.001
gower	81.858	0.001	19.159	0.001	7.751	0.001
altGower	78.690	0.001	44.500	0.001	4.001	0.002

Experiment indicates the individual communities that were constructed, Media indicates whether the community was grown in 1x or 0.1x BHI, and Condition indicates the adjusted ratios or equally mixed mode of community construction.

The constructed community when applied to an animal model can be successfully recovered with activity intact after 3 days indicative from the processed metagenomic and metatranscriptomic samples. Full potential for the use of the SkinCom has been detailed in Lekbua et al.^1^

## Limitations

The SkinCom constructed from 9 members is representative of the skin microbiome at the species level and can be expanded to include greater species or strain level diversity using the same protocol. The CellenONE X1 used for this protocol relies on the AutoDrop mode, which requires cultures to be pre-normalized to ensure appropriate 2-10 cells per droplet, however the variability can be higher if bacteria tend to be much larger in cell size or tend to clump/ form biofilms. Such characteristics can influence the reproducibility of SkinComs constructed with certain microbes.

## Troubleshooting

### Problem 1

Inconsistent droplet from CellenONE X1 pico-printer during priming.

### Potential solution

• The PDC and nozzle vicinity may be experiencing a lot of static. This can happen especially when using a lot of fresh plastic (like brand new 96-well corning plates). Use an anti-static gun (such as Milty Zerostat 3 Anti-Static Gun) around the PDC and the robotic arms while in “Continuous Dispense” mode.
• In case the nozzle is dirty from exterior dust or past experiments, the droplet prior to release from the PDC nozzle will face some capillary force, especially when the culture liquid is very dilute (like 0.1x BHI in our case) causing the droplet to break upon ejection. Remedy this using Bovine Serum Albumin (BSA) solution:
• Prepare fresh BSA by making 1% solution in pure H2O, centrifuge max speed 3 min to pellet any particles (or filter 0.22 μm).
• Add to the well of the probe plate.
• WashFlushStrong to clean.
• In ‘Do Task’, run TakeProbe10 μL.
• ‘Dip’ into water.
• Move to camera and run continuous drop; you should see drop shape improve over time.

### Problem 2

All 9 strains are not detected in the community when analyzing the DNA sequencing data.

### Potential solution

Insufficient DNA extraction during lysis. The community includes several gram-positive bacteria from the genus *Staphylococcus*. It is therefore essential to perform both mechanical lysis and chemical lysis thoroughly. The Vortex Adaptor used in step-by-step method details; step 24 is essential for this. If an appropriate Vortex Adaptor is not available, follow the manufacturer’s instructions for the settings to use with other equipment like a Tissue Homogenizer. Standardize the bead-beating duration and speeds for this equipment by using the ZymoBIOMICS Microbial Community DNA Standard (Cat# D6305) as a positive control. The community consists of a mix of gram negatives, gram positives and yeast, a complete, thorough extraction is obtained using the equipment at specific speeds when all members of the positive control community are also visible post sequencing. For more details, follow instructions from Zymobiomics.

### Problem 3

Droplet not dispensed during the cellenone_AutoDrop run (step 12).

### Potential solution

If the machine primes successfully with a detected droplet but fails to identify a droplet midway through the run, it is possible that the nozzle has clogged or has aspirated air, creating an air bubble. Remedy this with the following steps.

• Pause the run at this stage ensuring the community-construction progress is saved.
• Then, in ‘Do Task’, perform an “AirEx”. Ensure the nozzle has a uniform stream of droplets.
• If the drops coalesce near the ejection end of the nozzle, use the “NozzleWash” command (see Figure 1A) to dip the PDC into the wash stream to clear the static and reinstate a uniform droplet stream.
• After the run finishes, perform an “AutoDropDetect” at the camera station to see the drop restored.

The run can be resumed from where it left off. Refer to Methods video S2 for depiction of how the PDC clears the clog.

Methods video S2. Procedure for clearing the clog in the PDC using the “AirEx” command, related to troubleshooting problem 3

### Problem 4

Insufficient RNA recovered from swabs (steps 64-66).

### Potential solution

Store swab for DNA/RNA recovery in RNA Later to protect swab from RNases. Also use RNAse removal cleaning solution like RNAse-away to thoroughly wipe down benchtop, micropipettes, microcentrifuges and qPCR machines prior to use to further reduce RNAse contamination. Confirm purity of extracted RNA using a Nanodrop or using RINe number from Agilent TapeStation to confirm integrity of RNA.

## Resource availability

### Lead contact

Further information and requests for resources and reagents should be directed to and will be fulfilled by the lead contact, Karsten Zengler (kzengler@ucsd.edu).

### Technical contact

Technical questions on executing this protocol should be directed to and will be answered by the technical contact, Deepan Thiruppathy (dthirupp@ucsd.edu)

### Materials availability

This study did not generate any new material.

### Data and code availability

• New sequences have been deposited to the Sequence Read Archive with submission numbers SUB14342487 and SUB14347828 and BioProject number PRJNA1091705.
• All code used to process and analyze data is available on GitHub (https://github.com/alekbua/SkinCom) and has been archived via Zenodo (https://zenodo.org/records/14908419).
• Any additional information required to reanalyze the data reported in this paper is available from the lead contact upon request.

## Acknowledgments

We would like to thank Joshua Cantlon from SCIENION for providing guidance with setting up the CellenONE X1 and for training probes and targets for the machine. We also thank our collaborators Anna DiNardo and Kana Kuroki (UCSD) for performing a pilot study involving a murine model. We are thankful to Stephanie Flores Ramos (UCSD) for early work related to SkinCom assembly. Additionally, the authors would like to thank the past and present members of the Zengler Lab for their experimental contributions, lively discussion, and critical evaluation of this manuscript. This work was partly supported by 10.13039/501100017041Evonik Industries AG and the UC San Diego Center for Microbiome Innovation.

## Author contributions

K.Z., J.C., and C.M. conceptualized the study. D.T., A.L., J.C., F.A., A.K., C.M., M.T., and A.H. contributed to acquisition of data. D.T., A.L., J.C., Y.W., F.A., and A.K. contributed to data analysis. D.T., A.L., J.C., F.A., A.K., C.M., V.N., and K.Z. contributed to data interpretation. D.T., A.L., and K.Z. drafted the manuscript. D.T., A.L., J.C., F.A., A.K., V.N., and K.Z. revised the manuscript. K.Z. and V.N. acquired funding for the study. All authors read and approved the final version of the manuscript and had access to all the data in the study.

## Declaration of interests

The authors declare no competing interests.
